# The Prospective Dutch Colorectal Cancer (PLCRC) cohort: real-world data facilitating research and clinical care

**DOI:** 10.1038/s41598-020-79890-y

**Published:** 2021-02-16

**Authors:** Jeroen W. G. Derksen, Geraldine R. Vink, Marloes A. G. Elferink, Jeanine M. L. Roodhart, Helena M. Verkooijen, Wilhelmina M. U. van Grevenstein, Peter D. Siersema, Anne M. May, Miriam Koopman, The PLCRC Study Group, Geerard L. Beets, Eric J. Th. Belt, Maaike Berbée, Frederique H. Beverdam, Ruud Blankenburgh, Peter Paul L. O. Coene, Hester van Cruijsen, Jan Willem T. Dekker, Joyce M. van Dodewaard-de Jong, Frans L. G. Erdkamp, Jan Willem B. de Groot, Annebeth W. Haringhuizen, Helgi H. Helgason, Mathijs P. Hendriks, Ignace H. J. T. de Hingh, Ronald Hoekstra, Jan N. M. Ijzermans, Jan Jansen, Frank W. H. Kloppenberg, Anja U. G. van Lent, Maartje Los, Martijn R. Meijerink, Leonie J. M. Mekenkamp, Peter Nieboer, Koen C. M. J. Peeters, Natascha A. J. B. Peters, Marco B. Polée, Johannes F. M. Pruijt, Cornelis J. A. Punt, Patricia Quarles van Ufford-Mannesse, Ron C. Rietbroek, Anandi H. W. Schiphorst, Arjan Schouten van der Velden, Ruud W. M. Schrauwen, Mark P. S. Sie, Lieke Simkens, Dirkje W. Sommeijer, Dirk J. A. Sonneveld, Leontine E. A. Spierings, Hein B. A. C. Stockmann, Koen Talsma, Frederiek Terheggen, Albert J. ten Tije, Manuel L. R. Tjin-A-Ton, Liselot B. J. Valkenburg-van Iersel, Renzo P. Veenstra, Ankie M. T. van der Velden, Maarten Vermaas, Wouter J. Vles, Jeroen F. J. Vogelaar, Theo van Voorthuizen, Aad I. de Vos, Johannes A. Wegdam, Johannes H. W. de Wilt, David D. E. Zimmerman

**Affiliations:** 1 Department of Medical Oncology, University Medical Center Utrecht, Utrecht University, PO Box 85500, 3508 GA Utrecht, The Netherlands; 2 Julius Center for Health Sciences and Primary Care, University Medical Center Utrecht, Utrecht University, PO Box 85500, 3508 GA Utrecht, The Netherlands; 3 Department of Research, Netherlands Comprehensive Cancer Organisation (IKNL), PO Box 19079, 3501 DB Utrecht, The Netherlands; 4 Imaging Division, University Medical Center Utrecht, Utrecht University, PO Box 85500, 3508 GA Utrecht, The Netherlands; 5 Department of Surgical Oncology, University Medical Center Utrecht, Utrecht University, PO Box 85500, 3508 GA Utrecht, The Netherlands; 6 Department of Gastroenterology and Hepatology, University Medical Center Utrecht, Utrecht University, PO Box 85500, 3508 GA Utrecht, The Netherlands; 7 Department of Gastroenterology and Hepatology, Radboud UMC, Radboud University, PO Box 9101, 6500 HB Nijmegen, The Netherlands; 8 Department of Surgery, Nederlands Kanker Instituut - Antoni Van Leeuwenhoek Hospital, Amsterdam, The Netherlands; 9 Department of Surgery, Albert Schweitzer Hospital, Dordrecht, The Netherlands; 10 Department of Radiotherapy, Maastro Clinic, Maastricht, The Netherlands; 11 Department of Surgery, Franciscus Gasthuis and Vlietland Hospital, Schiedam, The Netherlands; 12 Department of Medical Oncology, Saxenburgh Hospital, Hardenberg, The Netherlands; 13 Department of Surgery, Maasstad Hospital, Rotterdam, The Netherlands; 14 Department of Medical Oncology, Antonius Hospital, Sneek, The Netherlands; 15 Department of Surgery, Reinier de Graaf Hospital, Delft, The Netherlands; 16 Department of Medical Oncology, Meander Medical Center, Amersfoort, The Netherlands; 17 Department of Medical Oncology, Zuyderland Hospital, Heerlen, The Netherlands; 18 Department of Medical Oncology, Isala Oncology Center, Zwolle, The Netherlands; 19 Department of Medical Oncology, Ziekenhuis Gelderse Vallei, Ede, The Netherlands; 20 Department of Medical Oncology, Haaglanden Medical Center, Den Haag, The Netherlands; 21 Department of Medical Oncology, Northwest Clinics, Alkmaar, The Netherlands; 22 Department of Surgery, Catharina Hospital, Eindhoven, The Netherlands; 23 Department of Medical Oncology, Ziekenhuisgroep Twente, Hengelo, The Netherlands; 24 Department of Surgery, Erasmus MC, University Medical Center Rotterdam, Rotterdam, The Netherlands; 25 Department of Surgery, Admiraal de Ruyter Hospital, Goes, The Netherlands; 26 Department of Surgery, Treant Hospital, Emmen, The Netherlands; 27 Department of Gastroenterology and Hepatology, Onze Lieve Vrouwe Hospital, Amsterdam, The Netherlands; 28 Department of Medical Oncology, St. Antonius Hospital, Nieuwegein, The Netherlands; 29 Department of Radiology and Nuclear Medicine, Amsterdam University Medical Center - Loc. VU, University of Amsterdam, Amsterdam, The Netherlands; 30 Department of Medical Oncology, Medisch Spectrum Twente, Enschede, The Netherlands; 31 Department of Medical Oncology, Wilhelmina Hospital, Assen, The Netherlands; 32 Department of Surgery, Leiden University Medical Center, University of Leiden, Leiden, The Netherlands; 33 Department of Medical Oncology, Sint Jans Hospital, Weert, The Netherlands; 34 Department of Medical Oncology, Medical Center Leeuwarden, Leeuwarden, The Netherlands; 35 Department of Medical Oncology, Jeroen Bosch Hospital, Den Bosch, The Netherlands; 36 Department of Medical Oncology, Amsterdam University Medical Center - Loc. AMC, University of Amsterdam, Amsterdam, The Netherlands; 37 Department of Medical Oncology, Haga Hospital, Den Haag, The Netherlands; 38 Department of Medical Oncology, Rode Kruis Hospital, Beverwijk, The Netherlands; 39 Department of Surgery, Diakonessenhuis Hospital, Utrecht, The Netherlands; 40 Department of Surgery, St Jansdal Hospital, Harderwijk, The Netherlands; 41 Department of Gastroenterology and Hepatology, Bernhoven Hospital, Uden, The Netherlands; 42 Department of Medical Oncology, ZorgSaam Hospital, Terneuzen, The Netherlands; 43 Department of Medical Oncology, Maxima Medical Center, Eindhoven, The Netherlands; 44 Department of Medical Oncology, Flevo Hospital, Almere, The Netherlands; 45 Department of Surgery, Dijklander Hospital, Purmerend, The Netherlands; 46 Department of Medical Oncology, Alrijne Hospital, Leiderdorp, The Netherlands; 47 Department of Surgery, Spaarne Hospital, Haarlem, The Netherlands; 48 Department of Surgery, Deventer Hospital, Deventer, The Netherlands; 49 Department of Medical Oncology, Bravis Hospital, Roosendaal, The Netherlands; 50 Department of Medical Oncology, Amphia Hospital, Breda, The Netherlands; 51 Department of Medical Oncology, Rivierenland Hospital, Tiel, The Netherlands; 52 Department of Medical Oncology, Maastricht University Medical Center, Maastricht, The Netherlands; 53 Department of Gastroenterology and Hepatology, Martini Hospital, Groningen, The Netherlands; 54 Department of Medical Oncology, Tergooi Hospital, Hilversum, The Netherlands; 55 Department of Surgery, Ijsselland Hospital, Capelle Aan Den Ijssel, The Netherlands; 56 Department of Surgery, Ikazia Hospital, Rotterdam, The Netherlands; 57 Department of Surgery, Viecuri Hospital, Venlo, The Netherlands; 58 Department of Medical Oncology, Rijnstate Hospital, Arnhem, The Netherlands; 59 Department of Medical Oncology, Van Weel-Bethesda Hospital, Dirksland, The Netherlands; 60 Department of Surgery, Elkerliek Hospital, Helmond, The Netherlands; 61 Department of Surgery, Radboud University Medical Center, University of Nijmegen, Nijmegen, The Netherlands; 62 Department of Surgery, Elisabeth-TweeSteden Hospital, Tilburg, The Netherlands

**Keywords:** Cancer epidemiology, Epidemiology, Outcomes research, Colorectal cancer, Medical research

## Abstract

Real-world data (RWD) sources are important to advance clinical oncology research and evaluate treatments in daily practice. Since 2013, the Prospective Dutch Colorectal Cancer (PLCRC) cohort, linked to the Netherlands Cancer Registry, serves as an infrastructure for scientific research collecting additional patient-reported outcomes (PRO) and biospecimens. Here we report on cohort developments and investigate to what extent PLCRC reflects the “real-world”. Clinical and demographic characteristics of PLCRC participants were compared with the general Dutch CRC population (n = 74,692, Dutch-ref). To study representativeness, standardized differences between PLCRC and Dutch-ref were calculated, and logistic regression models were evaluated on their ability to distinguish cohort participants from the Dutch-ref (AU-ROC 0.5 = preferred, implying participation independent of patient characteristics). Stratified analyses by stage and time-period (2013–2016 and 2017–Aug 2019) were performed to study the evolution towards RWD. In August 2019, 5744 patients were enrolled. Enrollment increased steeply, from 129 participants (1 hospital) in 2013 to 2136 (50 of 75 Dutch hospitals) in 2018. Low AU-ROC (0.65, 95% CI: 0.64–0.65) indicates limited ability to distinguish cohort participants from the Dutch-ref. Characteristics that remained imbalanced in the period 2017–Aug’19 compared with the Dutch-ref were age (65.0 years in PLCRC, 69.3 in the Dutch-ref) and tumor stage (40% stage-III in PLCRC, 30% in the Dutch-ref). PLCRC approaches to represent the Dutch CRC population and will ultimately meet the current demand for high-quality RWD. Efforts are ongoing to improve multidisciplinary recruitment which will further enhance PLCRC’s representativeness and its contribution to a learning healthcare system.

## Introduction

Global colorectal cancer (CRC) incidence is expected to increase in the coming decades^1^, which emphasizes the need to fulfill current knowledge gaps and improve clinical outcomes. In the current era of precision medicine, smaller treatment‐eligible target populations are both an advancement as well as a challenge in cancer research^2,3^. Due to the large amount of CRC subgroups defined by clinical characteristics in combination with the many (low-frequency) molecular markers^4–6^, the enrollment of sufficiently large sample sizes in studies evaluating the safety and efficacy of new therapeutic agents is a growing challenge. In addition, selective enrollment in most phase III randomized clinical trials (RCTs) may affect the generalizability of trial results and limits our understanding of the “true” treatment’s benefit-risk profile in the broader patient population. This is a constraint in clinical cancer research, given that international clinical guidelines are often based on results from strongly selected trial populations.

As advocated by both the research community and regulators such as the U.S. Food and Drug Administration (FDA) and the European Medicines Agency (EMA), the development of high quality population-based studies in cancer patients that provide real-world data (RWD) is a major research priority to overcome challenges in research methodologies, complement RCT data, and ultimately improve patient outcomes^7,8^. A learning healthcare system approach, defined as a circular system in which “science, informatics, incentives, and culture are aligned for continuous improvement and innovation, with best practices seamlessly embedded in the delivery process and new knowledge captured as an integral by-product of the delivery experience”^9^, uses RWD to accelerate knowledge generation and its translation into clinical practice. RWD mainly distinguishes itself from trial-based evidence by being population-level data originating from sources outside of the typical clinical research setting, such as electronic health records (EHRs) or cancer registries, with the potential to efficiently answer research questions relevant to the broader patient population^10^. To warrant high quality RWD, ascertaining a high quality of primary data (i.e. completeness and accuracy of EHRs), linkage of data sources, and quality of derived variables, is paramount^11,12^. Altogether, a prospective “real-world” cohort requires longitudinal patient, treatment (sequences), and outcome data from an unselected and representative patient population.

Since 2013, the Prospective Dutch Colorectal Cancer (PLCRC) cohort collects extensive longitudinal clinical data, together with blood, (tumor) tissue, and repeated patient-reported outcomes (PROs) in patients with stage I to IV CRC that are prospectively followed from primary diagnosis until death^13^. PLCRC serves as an infrastructure for a wide variety of research projects including etiological, biomarker, basic, (epi)genetic, and interventional [according to the Trials within Cohorts (TwiCs) design^14^], as well as health-care policy and cost-effectiveness studies. In order for results to be generalizable, and for accurate evaluation of cancer treatments, it is important to obtain a cohort that consists of a demographically and clinically representative patient population. Therefore, the aims of this manuscript are to (1) describe developments towards a nationwide cohort, (2) provide baseline characteristics, including PROs, of the first 5722 participants, and (3) investigate to what extent PLCRC reflects the “real-world”—over time and by tumor stage—by comparing PLCRC cohort participants with the general Dutch CRC population as registered in the Netherlands Cancer Registry (NCR).

## Methods

PLCRC is an initiative coordinated by the Dutch Colorectal Cancer Group (DCCG) and is registered at Clinicaltrials.gov (NCT02070146). The ‘Strengthening the Reporting of Observational Studies in Epidemiology (STROBE)’ guidelines were taken into account when the cohort was designed^15^. We here describe the cohort briefly since the detailed design is published elsewhere^13^.

### The PLCRC population

PLCRC consists of patients diagnosed with a malignancy of the colon and/or rectum (ICD-10, C18-20) in the Netherlands. Each patient with histologically proven, or a strong suspicion of CRC without pathological confirmation, who is ≥ 18 years of age is eligible. The informed consent procedure is preferably performed shortly after diagnosis and before treatment starts. However, patients can also be enrolled during treatment or follow-up. Consent for longitudinal clinical data collection is mandatory for participation. In addition, patients can choose to consent to other optional items as shown in Box 1. Patients enrolled before August 1, 2019 of whom complete clinical data of the initial diagnosis and treatment period were available—to ascertain correct classification of tumor stage—were included in the analyses. Of these patients, baseline demographic and clinical data were retrieved and reported, as well as self-reported physical activity, fatigue, quality of life, BMI, presence of chronic comorbidities, smoking behavior, alcohol consumption, education level, and living situation at baseline.

**Box 1:** Informed consent options and main objectives of PLCRC.

IC options within the PLCRC cohort
**Mandatory**
1. Informed consent for longitudinal observational clinical data collection
**Optional**
2. Informed consent for providing PRO (hard-copy or electronic) (a) In case of electronic patient-reported outcomes (ePRO), patients can choose to receive a summarized evaluation of their HRQoL (incl. optional comparisons with average scores of sex- and age-matched CRC patients and/or the non-cancer population) and share these data with their healthcare professional
3. Informed consent to approach the patient for future studies and to use their data in comparative research according to the Trials within Cohorts (TwiCs) design
4. Informed consent for biobanking of (tumor) tissue
5. Informed consent for providing additional blood samples during routine blood withdrawal for observational studies or biobanking
6. Informed consent to receive information in case of new relevant DNA abnormalities
**Objectives of the PLCRC cohort**
To prospectively collect detailed data on medical history, serious comorbidities, basic physical examination, imaging results, pathology results, tumor characteristics, treatments, treatment outcomes, hospital stays, interventions and adverse events
To collect blood and (tumor) tissue material, obtained during routine practice, for ongoing and future research
To provide more accurate treatment data, and clinical and patient-reported outcomes of CRC in daily clinical practice
To create a continuous basis for a large variety of research purposes including, but not limited to: Etiologic, diagnostic, and prognostic research Basic and (epi)genetic research Interventional studies according to TwiCs designs Healthcare policy and cost-effectiveness studies

### The Netherlands Cancer Registry (NCR)

The Netherlands Cancer Registry (NCR) contains an extensive set of clinical data—from diagnosis onwards—of individuals diagnosed with cancer in the Netherlands and has a national coverage of over 95%^16^. Clinical data of the complete treatment trajectory are retrieved from EHRs and entered into the NCR. Importantly, the completeness of the NCR therefore depends on the completeness of EHRs. Overall, the NCR’s high quality is assured by thorough training of data managers and computerized consistency checks. PLCRC’s informed consent allows for linkage with the NCR and thus ensures the availability of clinical data over the complete cancer trajectory.

For the current analysis, only data of the initial data registration phase, i.e. at diagnosis, were used. We compared characteristics of PLCRC participants with the general Dutch CRC population (Dutch-ref) from the NCR with incidences between January 1, 2013 and December 31, 2017.

### Statistical analysis

Descriptive statistics were used to describe baseline patient characteristics, including baseline PROs. Standardized differences (d) were calculated to quantify the magnitude of differences in patient characteristics between PLCRC participants and the Dutch-ref. Values greater than 0.20 indicate a large imbalance, while values between 0.10 and 0.20 indicate a small imbalance, and standardized differences less than 0.10 indicate a negligible imbalance^17,18^. Results are shown for the total group and stratified by tumor stage and time of enrollment. Two time-periods (enrolled between 2013–‘16 and 2017–August ’19) were evaluated to assess whether PLCRC participants became more representative of the Dutch-ref over time. Logistic regression models were used to investigate to what extent, based on the available a priori selected patient characteristics (i.e. age, sex, primary tumor location and tumor stage), cohort participation could be predicted^19^. Model performance was assessed based on calibration and discrimination^20^. Calibration—the goodness of fit—was evaluated using the Hosmer–Lemeshow test^21^. Discrimination refers to the ability to distinguish cohort participants from non-participants, and was quantified by the area under the receiver operating characteristic curve (AU-ROC)^20^. The AU-ROC ranges from 1, corresponding to perfect discrimination, to 0.5, corresponding to a model with no discrimination ability, here preferred and defined as cohort participation independent of patient characteristics (0.5 = random chance, 0.5–0.7 = poor, 0.7–0.8 = good, 0.8–1.0 = strong, 1.0 = perfect prediction)^22^. Statistical analyses were performed using STATA (Release 15, Stata Corp LLC, College Station, TX) and SPSS (version 25.0, IBM Corp, Armonk, NY).

### Ethics approval

The study protocol was approved by the Institutional Review Board of the University Medical Center Utrecht (The Netherlands). All procedures performed that involved human participants were in accordance with the institutional and/or national ethical standards and guidelines as well as with the 1964 Helsinki declaration and its later amendments or comparable ethical standards.

### Consent to participate

Informed consent was obtained from all individual participants included in the study.

## Results

**Figure 1:**
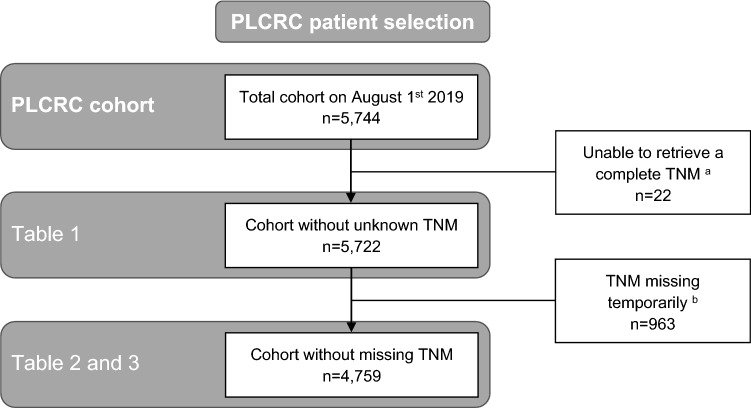
Flow diagram of the selection of individuals for the current analyses. ^a^Insufficient data in patient’s EHRs for the NCR to collect a complete TNM stage. ^b^Missing due to time-lag in NCR clinical data collection. This number is higher than presented in Table 1, as for some cases sufficient staging information was available to classify into TNM stage.

The flowchart for the selection of individuals for the current analyses is shown in Fig. 1. On August 1, 2019, a total of 5744 patients were enrolled. A complete TNM tumor stage could not be retrieved for 22 patients, who were therefore not included in the analyses.

### Developments towards a nationwide cohort

**Figure 2:**
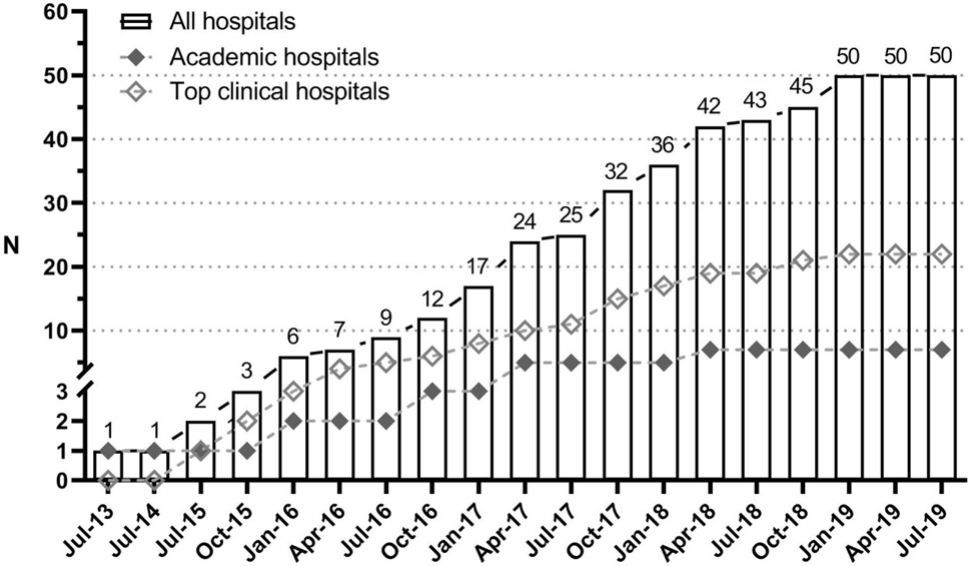
PLCRC recruiting hospitals (academic, non-academic, and top clinical hospitals) over time. Note: In The Netherlands there is a total of 75 hospitals, of which 8 academic hospitals and 26 top-clinical hospitals.

**Figure 3:**
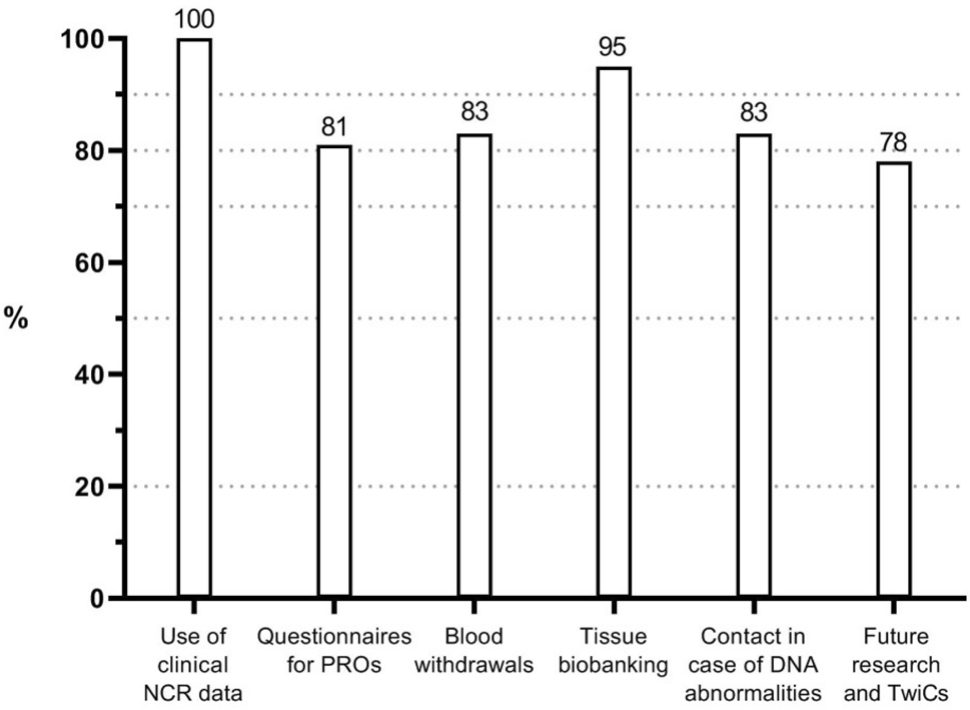
Baseline informed consent percentages per item. The use of clinical NCR data is 100% since this item is mandatory for participation. *NCR* Netherlands Cancer Registry, *PROs*  patient-reported outcomes, *TwiCs* Trials within Cohorts.

**Figure 4:**
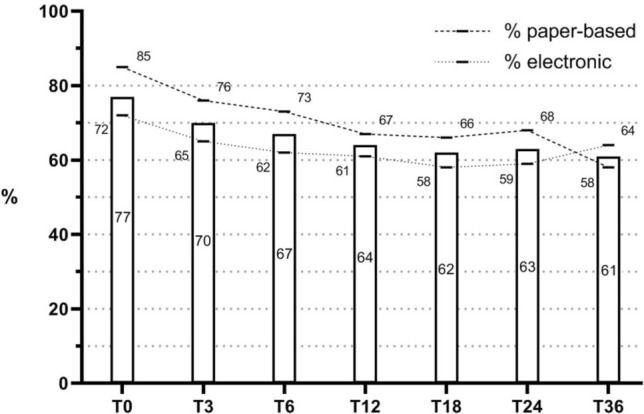
Completion rates of questionnaires until three years after enrollment. Overall completion rates are presented in the bars, and electronic and paper-based percentages at the dashed lines. Time-points (T) are months since enrollment.

PLCRC has continuously strengthened its infrastructure to improve the enrollment rate. In August 2019, patients were enrolled in 50 of 75 Dutch hospitals including 7 (of 8) academic hospitals, 22 (of 26) top clinical hospitals that focus on education and research, and 21 (of 41) regular hospitals (Fig. 2). This led to an improved annual enrollment-rate, from 129 participants from 1 recruiting hospital in 2013 to 2136 from 50 recruiting hospitals in 2018 (note that there are approx. 14,000 incident cases annually). At enrollment, 100% of patients consented for using their clinical data obtained from the NCR, which was mandatory, 81% consented to receive repeated PRO questionnaires, 83% for blood withdrawals, 95% for use of tissue for scientific research, 83% for contact when relevant DNA abnormalities are found, and 78% for future research and trials according to the TwiCs design (Fig. 3). Once consented to receive questionnaires for PROs, 77% of patients returned their baseline questionnaire, and completion rates remained above 60% in the first three years after enrollment (Fig. 4). Interestingly, patients who received paper-based questionnaires had consistently higher completion rates compared to electronic questionnaires (85% vs. 72% at baseline, respectively).

### Baseline patient characteristics

The cohort contained 851 patients with stage I CRC, 1079 with stage II, 1960 with stage III, 946 with stage IV, and 886 patients of whom data on tumor stage is still being collected (Table 1). The median number of days from diagnosis until enrollment was 18 (IQR: 1 to 131) for the total cohort, and was similar for all stages except for stage IV patients, who were enrolled much later in the cancer trajectory (188 [IQR: 23 to 670] days). The percentage of males was higher than females for all stages, and 61% of the patients had a primary tumor located in the colon, and 39% in the rectum. Of the 946 stage IV patients, 79% had synchronous liver, 22% lung, and 17% peritoneal metastases. Regarding molecular diagnostics at diagnosis, *RAS* status was available in 596 (10%) patients, *BRAF* in 570 (10%), and microsatellite instability (MSI) in 2600 (45%). In terms of physical and psychological wellbeing at enrollment (supplementary Table 1), patients reported to experience impaired psychosocial functioning, and high levels of fatigue, appetite loss and diarrhea as compared to reference populations (refs).

**Table 1:** Baseline descriptive demographic and clinical characteristics at PLCRC enrollment, stratified by tumor stage.

Baseline characteristics	Total^a^ (n = 5722)	Stage I (n = 851)	Stage II (n = 1079)	Stage III (n = 1960)	Stage IV (n = 946)	Stage missing^d^ (n = 886)
**Demographic and clinical characteristics**^**b**^						
**Year of enrollment (n = 5722)**						
2013–2016	1088 (19%)	160 (19%)	213 (20%)	462 (24%)	253 (27%)	0 (0%)
2017	1343 (24%)	240 (28%)	326 (33%)	512 (26%)	264 (28%)	1 (< 1%)
2018	2128 (37%)	369 (43%)	421 (39%)	759 (39%)	314 (33%)	265 (30%)
2019 (until August)	1163 (20%)	82 (10%)	119 (11%)	227 (12%)	115 (12%)	620 (70%)
**Age at enrollment (n = 5722)**	66.3 ± 10.6	67.7 ± 8.9	68.2 ± 10.3	65.7 ± 10.5	63.8 ± 10.9	66.7 ± 11.6
< 55 years	720 (13%)	49 (6%)	95 (9%)	263 (13%)	196 (21%)	117 (13%)
55–64 years	1652 (29%)	240 (28%)	290 (27%)	582 (30%)	286 (30%)	254 (29%)
65–74 years	2040 (36%)	372 (44%)	386 (36%)	708 (36%)	308 (33%)	266 (30%)
75–84 years	1117 (20%)	165 (19%)	249 (23%)	365 (19%)	140 (15%)	198 (22%)
≥ 85 years	193 (3%)	25 (3%)	59 (6%)	42 (2%)	16 (2%)	51 (6%)
**Days from diagnosis to enrollment (n = 5722)**	18 (1;131)	14 (0;45)	13 (-2;66)	23 (3;156)	188 (23;670)	4 (-10;20)
< 31 days	3397 (59%)	595 (70%)	719 (67%)	1087 (56%)	269 (28%)	727 (82%)
31–90 days	693 (12%)	109 (13%)	112 (10%)	255 (13%)	106 (11%)	111 (13%)
91–365 days	637 (11%)	61 (7%)	79 (7%)	239 (12%)	211 (22%)	47 (5%)
> 365 days	995 (17%)	86 (10%)	169 (16%)	379 (19%)	360 (38%)	1 (< 1%)
**Sex (n = 5722)**						
Male	3526 (62%)	532 (63%)	685 (64%)	1196 (61%)	583 (62%)	530 (60%)
Female	2196 (38%)	319 (38%)	394 (36%)	764 (39%)	363 (38%)	356 (40%)
**Primary tumor location (n = 5722)**						
Rectum (C19.9, C20.9)	2231 (39%)	321 (38%)	347 (32%)	1023 (52%)	364 (38%)	176 (20%)
Colon (C18.0–18.9)	3491 (61%)	530 (62%)	732 (68%)	937 (48%)	582 (62%)	710 (80%)
Right colon (C18.0–18.4)	1403 (40%)	254 (48%)	410 (56%)	432 (46%)	253 (44%)	54 (8%)
Left colon (C18.5–18.7)	1564 (45%)	270 (51%)	309 (42%)	489 (52%)	315 (54%)	181 (25%)
Colon unspecified (C18.8–18.9)	524 (15%)	6 (1%)	13 (2%)	16 (2%)	14 (2%)	475 (67%)
**Location synchronous metastases (n = 5722)**						
Liver	746 (13%)				746 (79%)	
Lung	212 (4%)				212 (22%)	
Peritoneal	163 (3%)				163 (17%)	
**Molecular diagnostics (n = 570–2600, missing 55–90%)**						
*RAS* mutation status determined	596 (10%)	9 (1%)	30 (3%)	119 (6%)	438 (46%)	0 (0%)
*BRAF* mutation status determined	570 (10%)	14 (2%)	48 (4%)	121 (6%)	387 (41%)	0 (0%)
Microsatellite instability (MSI) determined	2600 (45%)	436 (51%)	582 (54%)	1097 (56%)	483 (51%)	2 (< 1%)
**BMI at enrollment, kg/m**^**2**^** (n = 3108, missing 46%)**^**c**^	26.3 ± 4.5	27.1 ± 4.6	26.3 ± 4.6	26.1 ± 4.2	25.8 ± 5.0	26.2 ± 4.3
Underweight (< 18.5)	37 (1%)	2 (< 1%)	7 (1%)	11 (1%)	7 (1%)	10 (2%)
Normal weight (18.5–25)	1313 (42%)	170 (37%)	198 (39%)	466 (43%)	261 (48%)	218 (41%)
Overweight (25–30)	1254 (40%)	185 (40%)	214 (43%)	435 (40%)	199 (37%)	221 (42%)
Obese (> 30)	504 (16%)	101 (22%)	83 (17%)	165 (15%)	74 (14%)	81 (15%)
**Past 12 months treated for chronic disease, at enrollment (n = 2986, missing 48%)**^**c**^						
Cardiac disease or stroke	313 (11%)	59 (13%)	42 (9%)	114 (11%)	44 (9%)	54 (11%)
Diabetes	290 (10%)	38 (9%)	58 (12%)	117 (11%)	27 (5%)	50 (10%)
Liver disease	184 (6%)	11 (3%)	18 (4%)	35 (3%)	112 (22%)	8 (2%)
Kidney disease	38 (1%)	5 (1%)	6 (1%)	13 (1%)	7 (1%)	7 (1%)
**Smoking status at enrollment (n = 3140, missing 45%)**^**c**^						
Current	231 (7%)	23 (5%)	54 (11%)	74 (7%)	46 (8%)	34 (6%)
Former	1840 (59%)	293 (63%)	286 (56%)	646 (59%)	298 (55%)	317 (59%)
Never	1069 (34%)	149 (32%)	168 (33%)	368 (34%)	200 (37%)	184 (34%)
**Past month average alcohol consumption at enrollment (n = 3111, missing 46%)**^**c**^						
0 units per day	1167 (38%)	134 (29%)	165 (33%)	403 (37%)	259 (48%)	206 (39%)
0–1 units per day	1223 (39%)	195 (42%)	211 (42%)	400 (37%)	196 (36%)	221 (42%)
1–2 units per day	464 (15%)	82 (18%)	75 (15%)	182 (17%)	58 (11%)	67 (13%)
> 2 units per day	257 (8%)	50 (11%)	52 (10%)	94 (9%)	26 (5%)	35 (7%)
**Living situation at enrollment (n = 3144, missing 45%)**^**c**^						
Living alone	530 (17%)	74 (15%)	87 (17%)	183 (17%)	90 (17%)	96 (18%)
Living with partner, without children	1965 (63%)	324 (70%)	322 (63%)	679 (62%)	313 (57%)	327 (61%)
Living with partner and children	537 (17%)	51 (11%)	82 (16%)	189 (17%)	120 (22%)	95 (18%)
Living alone with children	69 (2%)	8 (2%)	12 (2%)	26 (2%)	12 (2%)	11 (2%)
Other	43 (1%)	8 (2%)	5 (1%)	13 (1%)	11 (2%)	6 (1%)
**Educational level (n = 3108, missing 46%)**^**c**^						
≤ High school	1206 (39%)	201 (44%)	194 (39%)	401 (37%)	192 (36%)	218 (41%)
Trade / college / other non-university	1548 (50%)	212 (46%)	256 (51%)	539 (50%)	278 (51%)	263 (49%)
University	354 (11%)	45 (10%)	53 (10%)	135 (13%)	70 (13%)	51 (10%)

Descriptives are presented as count (%), mean (± SD), or median (IQR).

^a^N = 22 patients with permanently unknown tumor stage are not included in the analysis.

^b^In case of missing data, the descriptive statistics of complete cases are presented.

^c^Self-reported.

^d^PLCRC is a dynamic cohort with continuous new enrollment and data-linkage. The high percentage of missing data, is due to the time-lag between enrollment and data linkage from the NCR to PLCRC, which is continuously updated.

### PLCRC versus the general Dutch CRC population

While the logistic regression model including age, sex, primary tumor location, and tumor stage overestimates the probability of participation (*p* < 0.001), the low discriminative power (AU-ROC 0.65, 95% CI: 0.64–0.65) indicates limited ability to distinguish cohort participants from the Dutch-ref, based on available data (full ROC curves in supplementary Fig. 1). This discrimination decreased over time from an AU-ROC of 0.70 (95% CI: 0.68–0.71) in PLCRC’s initial phase (2013–‘16) to 0.64 (95% CI: 0.63–0.64) in the most recent phase (2017–Aug ‘19).

Between PLCRC participants (n = 4759) and the Dutch-ref (n = 72,685), large imbalances were found for age at diagnosis (64.9 years in PLCRC, 69.3 in the Dutch-ref, d_age_ 0.41), primary tumor location (43% rectum in PLCRC and 31% in the Dutch-ref, d_pr.tumor_ 0.24) and TNM stage (41% stage III in PLCRC and 30% in the Dutch-ref, d_tnm_ 0.24), a small imbalance in sex (62% male in PLCRC and 57% in the Dutch-ref, d_sex_ 0.11), and negligible imbalances in BMI at diagnosis (26.6 in PLCRC and 26.6 in the Dutch-ref, d_bmi_ 0.01) and in location of synchronous metastasis (15% liver in PLCRC and 15% in the Dutch-ref, d_meta_ between 0.01 and 0.08), Table 2.

**Table 2:** Characteristics of PLCRC participants at diagnosis (2013-Aug’19), compared with the general Dutch CRC population (2013–’17), and stratified by time-period (2013–’16, and 2017–Aug’19).

Baseline characteristics	Dutch population with CRC between 2013- ‘17 (n = 72,685)	All PLCRC participants 2013-Aug’19 (n = 4759)	Standardized difference (d)^a^	PLCRC’s initial phase 2013-’16 (n = 1088)	Standardized difference (d)^a^	PLCRC’s most recent phase 2017-Aug’19 (n = 3671)	Standardized difference (d)^a^
**Age at diagnosis**	69.3 ± 10.8	64.9 ± 10.5	0.41	64.6 ± 10.2	0.45	65.0 ± 10.6	0.40
< 55 years	6476 (9%)	733 (15%)		170 (16%)		563 (15%)	
55–64 years	15,248 (21%)	1470 (31%)		350 (32%)		1120 (31%)	
65–74 years	26,602 (37%)	1722 (36%)		395 (36%)		1327 (36%)	
75–84 years	19,482 (27%)	729 (15%)		153 (14%)		576 (16%)	
≥ 85 years	4877 (7%)	105 (2%)		20 (2%)		85 (2%)	
**Sex**			0.11		0.17		0.09
Male	41,115 (57%)	2949 (62%)		704 (65%)		2245 (61%)	
Female	31,570 (43%)	1810 (38%)		384 (35%)		1426 (39%)	
**BMI at diagnosis**^**b**^** (kg/m**^**2**^**)**	26.6 ± 4.6	26.6 ± 4.8	0.01	25.9 ± 4.0	0.16	26.7 ± 4.9	0.03
Underweight (< 18.5)	228 (2%)	10 (1%)		1 (1%)		9 (1%)	
Normal weight (18.5–24.9)	5252 (38%)	502 (40%)		78 (47%)		424 (39%)	
Overweight (25–29.9)	5625 (41%)	503 (40%)		61 (37%)		442 (41%)	
Obese (≥ 30)	2691 (20%)	229 (18%)		26 (16%)		203 (19%)	
**Primary tumor location**^**c**^			0.24		0.53		0.16
Rectum (C19.9, C20.9)	22,426 (31%)	2025 (43%)		610 (56%)		1415 (39%)	
Colon (C18.0–18.7)	50,259 (69%)	2734 (57%)		478 (44%)		2256 (61%)	
Right colon (C18.0–18.4)	24,244 (48%)	1330 (49%)		200 (42%)		1130 (50%)	
Left colon (C18.5–18.7)	24,634 (49%)	1359 (50%)		269 (56%)		1090 (48%)	
Colon unspecified (C18.8–18.9)	1381 (3%)	45 (2%)		9 (2%)		36 (2%)	
**TNM**			0.24		0.33		0.22
Stage I	17,686 (24%)	849 (18%)		160 (15%)		689 (19%)	
Stage II	17,951 (25%)	1063 (22%)		213 (20%)		850 (23%)	
Stage III	21,707 (30%)	1929 (41%)		462 (42%)		1467 (40%)	
Stage IV	15,341 (21%)	918 (19%)		253 (23%)		665 (18%)	
**Location synchronous metastases**							
Liver	11,218 (15%)	722 (15%)	0.01	202 (19%)	0.08	520 (14%)	0.04
Lung	3954 (5%)	209 (4%)	0.05	58 (5%)	0.00	151 (4%)	0.06
Peritoneal	3673 (5%)	162 (3%)	0.08	42 (4%)	0.06	120 (3%)	0.09

^a^Standardized differences (d) are differences in means or proportions divided by standard error; d > 0.20 indicate a large difference, d 0.10–0.20 indicate a small difference, and d < 0.10 indicate a negligible difference^17,18^.

^b^BMI at diagnosis for PLCRC only available when participants were enrolled at diagnosis and provided PROs (n = 1244); the NCR only collected height and weight in 2015, thus the reference values for BMI originate only from patients diagnosed in 2015 (n = 13,796).

^c^Standardized differences calculated over the proportion rectum versus colon tumors.

When the two time-periods were compared with the Dutch-ref to study PLCRC’s evolution, a large imbalance remained for age at diagnosis and tumor stage (d_age_ from 0.45 to 0.40, d_tnm_ from 0.33 to 0.22). The distribution of sex, BMI at diagnosis, and primary tumor location improved to imbalances classified as small or negligible (d_sex_ from 0.17 to 0.09, d_bmi_ from 0.16 to 0.03, d_pr.tumor_ from 0.53 to 0.16). For location of synchronous metastases, e.g. liver metastasis, the imbalance compared with the Dutch-ref was negligible in both time periods (d_liver_ from 0.08 to 0.04).

Table 3 shows stratified analyses in which PLCRC participants were compared with the Dutch-ref by tumor stage. Age at diagnosis was lower for PLCRC participants in all stages, and discrepancies increased by stage (d_age_ from 0.28 in stage I to 0.55 in stage IV). For all disease stages, PLCRC contained relatively more patients with a primary tumor in the rectum and fewer patients with a primary tumor in the colon, compared with the Dutch-ref (d_pr.tumor_ between 0.14 and 0.25). The proportions of sex and BMI at diagnosis were comparable to the ref. population in all stages (d_sex_ between 0.08 and 0.19, d_bmi_ between 0.01 and 0.08).

**Table 3:** Characteristics of the PLCRC participants at diagnosis (2013–Aug’19), compared with the general Dutch CRC population (2013–’17), stratified by tumor stage.

Baseline characteristics	Dutch population with CRC between 2013- ‘17 Stage I (n = 17,686)	PLCRC participants 2013-Aug’19 Stage I (n = 849)	Standardized difference (d)^a^	Dutch population with CRC between 2013- ‘17 Stage II (n = 17,951)	PLCRC participants 2013-Aug’19 Stage II (n = 1063)	Standardized difference (d)^a^	Dutch population with CRC between 2013- ‘17 Stage III (n = 21,707)	PLCRC participants 2013-Aug’19 Stage III (n = 1929)	Standardized difference (d)^a^	Dutch population with CRC between 2013- ‘17 Stage IV (n = 15,341)	PLCRC participants 2013-Aug’19 Stage IV (n = 918)	Standardized Difference (d)^a^
**Age at diagnosis**	69.3 ± 9.1	66.8 ± 9.0	0.28	71.2 ± 10.6	66.9 ± 10.4	0.41	68.4 ± 11.2	64.2 ± 10.6	0.38	68.4 ± 11.8	62.2 ± 11.0	0.55
< 55 years	910 (5%)	64 (8%)		1205 (7%)	123 (12%)		2440 (11%)	326 (17%)		1921 (13%)	220 (24%)	
55–64 years	3.861 (22%)	258 (30%)		3117 (17%)	302 (28%)		4929 (23%)	607 (31%)		3341 (22%)	303 (33%)	
65–74 years	7.486 (42%)	367 (43%)		6362 (35%)	385 (36%)		7656 (35%)	689 (36%)		5098 (33%)	281 (31%)	
75–84 years	4.674 (26%)	143 (17%)		5612 (31%)	211 (20%)		5341 (25%)	273 (14%)		3855 (25%)	102 (11%)	
≥ 85 years	755 (4%)	17 (2%)		1655 (9%)	42 (4%)		1341 (6%)	34 (2%)		1126 (7%)	12 (1%)	
**Sex**			0.08			0.19			0.08			0.11
Male	10,411 (59%)	531 (63%)		9695 (54%)	675 (64%)		12,340 (57%)	1176 (61%)		8669 (57%)	567 (62%)	
Female	7275 (41%)	318 (37%)		8256 (46%)	388 (36%)		9367 (43%)	753 (39%)		6672 (43%)	351 (38%)	
**BMI at diagnosis**^**b**^** (kg/m**^**2**^**)**	27.2 ± 4.6	27.2 ± 4.8	0.01	26.5 ± 4.7	26.9 ± 5.0	0.08	26.6 ± 4.6	26.3 ± 4.4	0.08	25.6 ± 4.4	25.9 ± 5.7	0.06
Underweight (< 18.5)	28 (1%)	1 (0%)		72 (2%)	4 (1%)		62 (1%)	5 (1%)		66 (3%)	0 (0%)	
Normal weight (18.5–24.9)	1134 (32%)	119 (40%)		1323 (39%)	103 (35%)		1606 (37%)	216 (42%)		1189 (46%)	64 (50%)	
Overweight (25–29.9)	1532 (44%)	112 (37%)		1367 (40%)	129 (43%)		1774 (41%)	214 (41%)		952 (37%)	48 (37%)	
Obese (≥ 30)	798 (23%)	68 (23%)		668 (19%)	61 (21%)		855 (20%)	83 (16%)		370 (14%)	17 (13%)	
**Primary tumor localization**^**c**^			0.14			0.25			0.24			0.23
Rectum (C19.9, C20.9)	5543 (31%)	321 (38%)		3836 (21%)	342 (32%)		8813 (41%)	1008 (52%)		4234 (28%)	354 (39%)	
Colon (C18.0–18.7)	12,143 (69%)	528 (62%)		14,115 (79%)	721 (68%)		12,894 (59%)	921 (48%)		11,107 (72%)	564 (61%)	
Right colon (C18.0–18.4)	4686 (39%)	253 (48%)		7757 (55%)	406 (56%)		6314 (49%)	425 (46%)		5487 (49%)	246 (44%)	
Left colon (C18.5–18.7)	7245 (60%)	269 (51%)		6040 (43%)	304 (42%)		6326 (49%)	481 (52%)		5023 (45%)	305 (54%)	
Colon unspecified (C18.8–18.9)	212 (2%)	6 (1%)		318 (2%)	11 (2%)		254 (2%)	15 (2%)		597 (5%)	13 (2%)	

^a^Standardized differences (d) are differences in means or proportions divided by standard error; d > 0.20 indicate a large difference, d 0.10–0.20 indicate a small difference, and d < 0.10 indicate a negligible difference^17,18^.

^b^BMI at diagnosis for PLCRC only available when participants were enrolled at diagnosis and provided PROs (n = 1244); the NCR only collected height and weight in 2015, thus the reference values for BMI originate only from patients diagnosed in 2015 (n = 13,796).

^c^Standardized differences calculated over the proportion rectum versus colon tumors.

## Discussion

Over the past six years, the increased number of PLCRC recruiting centers has resulted in a steep increase in participating patients, with excellent consent rates for PROs, blood and tissue biobanking, and participation in future research within PLCRC. Although we found an overall shift towards the Dutch-ref for patients enrolled between 2017–Aug 2019, regular hospitals remain underrepresented as participating centers to enroll patients and PLCRC participants were still younger and more often had stage III disease, as compared to the total Dutch CRC population.

Besides common discrepancies such as performance status and number of comorbidities, clinical trial participants are notably younger than the real-world population, which for now might hamper the applicability of trial results in daily clinical practice. It was recently shown that phase-III RCT patients were on average seven years younger than the general CRC population^23^. Similarly, patients within PLCRC are younger compared to the Dutch-ref, however, this difference only is 4 years (mean age 65 years). Standardized differences for age increased by tumor stage, with stage IV patients showing a mean age comparable to phase-III clinical trials in metastatic CRC^24^. This emphasizes the need to focus on the enrollment of (stage IV) patients that are diagnosed at older age. Although important factors such as comorbidities and performance status are currently unknown, we believe that PLCRC has the potential to serve as a research platform that fulfills the current demand for RWD as advocated for by regulators and research community. The additional advantage of PLCRC is the large collection of biospecimen, which is intertwined with routine clinical care, and longitudinal PROs from diagnosis onwards. Moreover, the incorporation of PROs that describe the impact of treatment on quality of life, daily activities and symptoms is increasingly recognized as an essential component of real-world evidence and has the potential to improve cancer care, shared decision making, and clinical outcomes^25–27^.

Dutch CRC guidelines recommend, in line with the European guidelines, to determine both mismatch repair status in stage II-IV tumors, and *RAS* and *BRAF* mutation status in tumor of patients with metastatic CRC prior to the start of systemic treatment^28–30^. Although our percentages may be an underestimation as mutation status could become available during NCR updates after the initial data registration, the amount of missing data on molecular diagnostics is noteworthy. A limitation that is currently inevitable within PLCRC is that completeness of the NCR depends on daily clinical practices. In contrast to the above mentioned national guidelines, molecular markers are not routinely measured in all patients in the clinic. This means that currently, PLCRC is missing opportunities to optimally use tumor mutation status for research purposes. Efforts are ongoing to perform retrospective molecular profiling within PLCRC to supplement existing molecular pathology data with the aim to be able to tailor treatment options to the individual patient in the future. Next to the identification of predictors for treatment response and clinical outcomes, this will also contribute to the development of a unique cohort that could provide “external” controls for future single arm clinical trials in uncommon CRC subtypes with high unmet medical need^31,32^.

Given the large variety of available data, PLCRC will allow for comprehensive analyses on CRC. However, future improvements are required to optimize two fundamental elements of RWD sources: completeness of cases and completeness of clinical data. Based on our experience, over 90% of patients provide informed consent once the study aim is explained. Enhanced integration of research into daily clinical practice and the development of local infrastructures that lead to increased willingness and availability of personnel to inform the patient about PLCRC, especially in regular hospitals, are crucial to further improve the completeness of cases and create a true RWD cohort. Second, completeness of clinical data mainly depends on how well clinicians document clinical data in EHRs. Regardless of the list of items to be collected in the NCR, unmeasured or undocumented data will never become available to the research community. Moreover, EHR data are often unstructured and inconsistent due to large variation between clinicians and differences in EHR software systems. Bertagnolli and colleagues^33^ recently stated that the use of data obtained during routine clinical care as “real-world” data to fuel a learning healthcare system is currently still in its infancy. Prior to utilizing EHRs to facilitate a learning health system, EHRs must contain readily exchangeable and clinically meaningful structured data elements of adequate quality to draw valid inferences^33^. Therefore, we emphasize that nationwide harmonization and standardization of clinical data entries in EHRs and subsequent implementation of electronic data-capture systems to enable real-time data transfer from EHRs to the NCR, will significantly enhance the completeness and quality of clinical data.

Future focus should be given to reaching and enrolling older patients and to enhance involvement of the gastroenterology departments to enroll patients with early stage tumors. Moreover, especially stage IV patients should be enrolled closer to diagnosis to standardize time points for PROs and avoid potential survivor bias. This can be achieved by an optimal research-focused infrastructure and implementation of research-specific consultations for all cancer patients shortly after diagnosis. During this consultation, the patient is informed about the specific components of PLCRC (Box 1), as well as on the main aim to optimally evaluate treatments, accelerate innovation, and learn from each individual patient. Such an infrastructure will also contribute to enrolling patients with the least hospital visits, e.g. patients with a polypectomy only, or extensively metastasized disease with rapid progression and best supportive care only. Besides the aforementioned suggestions, we need to create a societal change with respect to clinical research. All stakeholders should be aware that, in order to improve oncology practice, research needs to become an integrated part of clinical care and that contributions to clinical research are self-evident. Lastly, PLCRC is a platform to centralize national CRC research to maximize its potential and minimize patient burden. Access to cohort resources for collaborative research projects may be requested through the Scientific Committee [https://plcrc.nl/for-international-visitors] that reviews all research projects for approval.

To conclude, PLCRC is establishing a unique and steeply growing national RWD cohort that allows for a wide range of research. Data from the general patient population enables a learning healthcare system that provides insight into the care and outcomes of patients that are usually underrepresented in RCTs, e.g. the very young and older patients and the ones with multiple comorbidities. Comprehensive analyses within PLCRC are facilitated by the extensive amount of clinical data covering the complete treatment trajectory and additional patient-reported outcomes. Further improvements in recruitment methodologies and multidisciplinary enrollment of patients will contribute to the aim of enrolling all newly diagnosed CRC patients in the Netherlands. This will continue to enhance PLCRC’s representation of the real-world and its ability to improve both scientific research and daily clinical practice.

## Supplementary information

Supplementary Information.

## Data Availability

Access to cohort resources for collaborative research projects may be requested through the Scientific Committee [https://plcrc.nl/for-international-visitors] that reviews all research projects for approval.

## References

[1] Arnold M (2017). Global patterns and trends in colorectal cancer incidence and mortality. Gut.

[2] Simon R (2015). The role of nonrandomized trials in the evaluation of oncology drugs. Clin. Pharmacol. Ther..

[3] Klauschen F, Andreeff M, Keilholz U, Dietel M, Stenzinger A (2014). The combinatorial complexity of cancer precision medicine. Oncoscience.

[4] Dekker E, Tanis PJ, Vleugels JLA, Kasi PM, Wallace MB (2019). Colorectal cancer. Lancet.

[5] Cancer Genome Atlas N (2012). Comprehensive molecular characterization of human colon and rectal cancer. Nature.

[6] Lee MS, Menter DG, Kopetz S (2017). Right versus left colon cancer biology: Integrating the consensus molecular subtypes. J. Natl. Compr. Cancer Netw..

[7] Klonoff DC (2019). The new FDA real-world evidence program to support development of drugs and biologics. J. Diabetes Sci. Technol..

[8] Eichler HG (2019). Data rich, information poor: Can we use electronic health records to create a learning healthcare system for pharmaceuticals?. Clin. Pharmacol. Ther..

[9] Institute of Medicine (2011). Engineering a Learning Healthcare System: A Look at the Future: Workshop Summary.

[10] Sherman RE (2016). Real-world evidence—What is it and what can it tell us?. N. Engl. J. Med..

[11] Booth CM, Karim S, Mackillop WJ (2019). Real-world data: Towards achieving the achievable in cancer care. Nat. Rev. Clin. Oncol..

[12] Cook JA, Collins GS (2015). The rise of big clinical databases. Br. J. Surg..

[13] Burbach JP (2016). Prospective Dutch colorectal cancer cohort: An infrastructure for long-term observational, prognostic, predictive and (randomized) intervention research. Acta Oncol..

[14] Relton C, Torgerson D, O'Cathain A, Nicholl J (2010). Rethinking pragmatic randomised controlled trials: Introducing the “cohort multiple randomised controlled trial” design. BMJ.

[15] von Elm E (2007). The strengthening the reporting of observational studies in epidemiology (STROBE) statement: Guidelines for reporting observational studies. Lancet.

[16] Schouten LJ, Hoppener P, van den Brandt PA, Knottnerus JA, Jager JJ (1993). Completeness of cancer registration in Limburg, The Netherlands. Int. J. Epidemiol..

[17] Cohen J (1962). The statistical power of abnormal-social psychological research: A review. J. Abnorm. Soc. Psychol..

[18] Austin PC (2009). Using the standardized difference to compare the prevalence of a binary variable between two groups in observational research. Commun. Stat. Simul. Comput..

[19] Steyerberg EW (2010). Assessing the performance of prediction models a framework for traditional and novel measures. Epidemiology.

[20] Harrell FE, Lee KL, Mark DB (1996). Multivariable prognostic models: Issues in developing models, evaluating assumptions and adequacy, and measuring and reducing errors. Stat. Med..

[21] Hosmer DW, Hosmer T, leCessie S, Lemeshow S (1997). A comparison of goodness-of-fit tests for the logistic regression model. Stat. Med..

[22] Hosmer D, Lemeshow S (2000). Applied Logistic Regression.

[23] Ludmir EB (2019). Factors associated with age disparities among cancer clinical trial participants. JAMA Oncol..

[24] Renfro LA (2016). Body mass index is prognostic in metastatic colorectal cancer: Pooled analysis of patients from first-line clinical trials in the ARCAD database. J. Clin. Oncol..

[25] Bottomley A (2019). Current state of quality of life and patient-reported outcomes research. Eur. J. Cancer.

[26] Nipp RD, Temel JS (2018). Harnessing the power of patient-reported outcomes in oncology. Clin. Cancer. Res..

[27] Calvert MJ, O'Connor DJ, Basch EM (2019). Harnessing the patient voice in real-world evidence: The essential role of patient-reported outcomes. Nat. Rev. Drug Discov..

[28] Oncoline. *Dutch Guidelines for Colorectal Cancer* https://www.oncoline.nl/colorectaalcarcinoom (2019).

[29] Labianca R (2013). Early colon cancer: ESMO Clinical Practice Guidelines for diagnosis, treatment and follow-up. Ann. Oncol..

[30] Van Cutsem E (2016). ESMO consensus guidelines for the management of patients with metastatic colorectal cancer. Ann. Oncol..

[31] Khozin S, Blumenthal GM, Pazdur R (2017). Real-world data for clinical evidence generation in oncology. J. Natl. Cancer Inst..

[32] Corrigan-Curay J, Sacks L, Woodcock J (2018). Real-world evidence and real-world data for evaluating drug safety and effectiveness. JAMA.

[33] Bertagnolli MM (2020). Status update on data required to build a learning health system. J. Clin. Oncol..

