# Supplementary material for: The Prospective Dutch Colorectal Cancer (PLCRC) cohort: real-world data facilitating research and clinical care
Source: Sci Rep. 2021 Feb 16;11:3923. doi: 10.1038/s41598-020-79890-y (PMC7887218; doi:10.1038/s41598-020-79890-y)
Supplement: Supplementary file 1 — Supplementary Information. [file 41598_2020_79890_MOESM1_ESM.docx]

**The Prospective Dutch Colorectal Cancer (PLCRC) Cohort: “real-world” data facilitating research and clinical care**

Jeroen W.G. Derksen, Ph.D. ^1,2^, Geraldine R. Vink, MD, Ph.D. ^1,3^, Marloes A.G. Elferink, Ph.D. ^3^, Jeanine M.L. Roodhart, MD, Ph.D. ^1^, Helena M. Verkooijen, Ph.D. ^4^, Wilhelmina M.U. van Grevenstein, MD, Ph.D. ^5^, Peter D. Siersema, MD, Ph.D. ^6,7^, Anne M. May, Ph.D. ^2^ †, Miriam Koopman, MD, Ph.D. ^1^ † *, “the PLCRC study group".

*† : shared last authorship*

1) Department of Medical Oncology, University Medical Center Utrecht, Utrecht University, PO Box 85500, 3508 GA, Utrecht, The Netherlands.

2) Julius Center for Health Sciences and Primary Care, University Medical Center Utrecht, Utrecht University, PO Box 85500, 3508 GA, Utrecht, The Netherlands.

3) Department of Research, Netherlands Comprehensive Cancer Organisation (IKNL), PO Box 19079, 3501 DB, Utrecht, the Netherlands.

4) Imaging Division, University Medical Center Utrecht, Utrecht University, PO Box 85500, 3508 GA, Utrecht, The Netherlands.

5) Department of Surgical Oncology, University Medical Center Utrecht, Utrecht University, PO Box 85500, 3508 GA, Utrecht, The Netherlands.

6) Department of Gastroenterology and Hepatology, University Medical Center Utrecht, Utrecht University, PO Box 85500, 3508 GA, Utrecht, The Netherlands.

7) Department of Gastroenterology and Hepatology, Radboud UMC, Radboud University, PO Box 9101, 6500 HB, Nijmegen, The Netherlands.

**Supplementary Table 1**. Mean scores (± standard deviations) of patient-reported physical activity, fatigue, and quality of life of all respondents at any time of PLCRC enrollment, and in a subgroup enrolled at diagnosis.

|  | PLCRC participants  at any time of enrollment  (n=4,759) | | PLCRC participants  enrolled at diagnosis  (n=2,615) | | Reference population * | |
| --- | --- | --- | --- | --- | --- | --- |
|  |  | |  | |  | |
| Physical activity (SQUASH) | n | Mean ± SD | n | Mean ± SD | n | Mean ± SD |
| Light intensity (<3 MET) in min/wk | 2,481 | 1,298 ± 1,048 | 1,220 | 1,396 ± 1,093 |  |  |
| Moderate intensity (3-6 MET) in min/wk | 2,481 | 582 ± 695 | 1,220 | 662 ± 762 |  |  |
| Vigorous intensity (≥6 MET) in min/wk | 2,481 | 24 ± 90 | 1,220 | 31 ± 101 |  |  |
| Adherence to Dutch guideline ‡ | 2,481 | 893 (36%) | 1,220 | 486 (40%) | 3,527 | 1,633 (46%) |
| Fatigue (MFI-20, range 4-20) |  | |  | |  | |
| General fatigue | 2,494 | **11.2 ± 4.9** | 1,220 | 10.6 ± 4.9 | 2,037 | 8.7 ± 3.6 |
| Physical fatigue | 2,496 | **10.9 ± 4.8** | 1,221 | 10.3 ± 4.8 | 2,037 | 8.4 ± 4.1 |
| Reduced activity | 2,489 | **11.4 ± 4.6** | 1,216 | **11.1 ± 4.6** | 2,037 | 8.0 ± 3.3 |
| Reduced motivation | 2,491 | 9.8 ± 4.1 | 1,221 | 9.6 ± 4.1 | 2,037 | 8.4 ± 3.8 |
| Mental fatigue | 2,495 | 8.7 ± 4.2 | 1,219 | 8.5 ± 4.1 | 2,037 | 7.7 ± 3.3 |
| Health-related quality of life  (QLQ-C30, range 0-100) |  | |  | |  | |
| Functional scales |  | |  | |  | |
| Overall quality of life | 2,854 | 71 ± 20 | 1,440 | 73 ± 19 | 1,731 | 78 ± 17 |
| Physical functioning | 2,861 | 86 ± 17 | 1,445 | 88 ± 16 | 1,731 | 90 ± 15 |
| Role functioning | 2,857 | **74 ± 30** | 1,442 | **78 ± 30** | 1,731 | 89 ± 21 |
| Emotional functioning | 2,859 | 80 ± 19 | 1,444 | 80 ± 19 | 1,731 | 89 ± 16 |
| Cognitive functioning | 2,859 | 87 ± 18 | 1,444 | 88 ± 17 | 1,731 | 92 ± 15 |
| Social functioning | 2,859 | **80 ± 23** | 1,444 | **83 ± 22** | 1,731 | 94 ± 16 |
| Symptoms |  | |  | |  | |
| Fatigue | 2,858 | **29 ± 25** | 1,444 | 26 ± 25 | 1,731 | 17 ± 20 |
| Nausea and vomiting | 2,859 | 7.1 ± 16 | 1,444 | 6.2 ± 16 | 1,731 | 2.7 ± 10 |
| Pain | 2,861 | 17 ± 24 | 1,445 | 16 ± 23 | 1,731 | 15 ± 22 |
| Dyspnea | 2,851 | 12 ± 21 | 1,441 | 10 ± 20 | 1,731 | 7.1 ± 17 |
| Insomnia | 2,858 | 23 ± 29 | 1,444 | 23 ± 29 | 1,731 | 14 ± 23 |
| Appetite loss | 2,857 | **14 ± 25** | 1,443 | 12 ± 24 | 1,731 | 3.3 ± 12 |
| Constipation | 2,851 | 11 ± 21 | 1,442 | 12 ± 22 | 1,731 | 4.8 ± 14 |
| Diarrhea | 2,846 | **18 ± 26 †** | 1,438 | **19 ± 27 †** | 1,731 | 3.9 ± 14 |

**Caption:** Baseline PROs for the complete cohort of respondents, and for a subset of patients enrolled at diagnosis (<31 days), obtained from the EORTC QLQ-C30, SCQ, SQUASH, and the MFI-20^1-4^. To put PROs into perspective, outcomes were evaluated based on pre-determined minimal clinically relevant differences relative to normative data, i.e. 2 points difference in fatigue^5^ and 10 points difference in HRQoL^6^. Clinically relevant difference are shown in bold.
Absolute HRQoL scores were also evaluated based on recently published thresholds for identification of clinically important symptoms and functional health impairments^7^.
† Above the absolute threshold for clinically important impairments.
* Reference physical activity levels originate from Statistics Netherlands^8^ (to obtain a reference group of comparable age, subjects with an age ranging between the 5th and 95th percentile of the age within the PLCRC cohort [i.e. 48-83 years] were selected and individuals living with and beyond cancer were excluded), reference values for fatigue originate from Schwarz *et al.*^9^, and HRQoL from van de Poll-Franse *et al.*^10^.
‡ 2017 Dutch Physical Activity Guideline^11^. Note: HRQoL scores and standard deviations ≥ 10 are rounded.
MET = metabolic equivalent of task (1 MET is equivalent to the consumption of 3.5 ml of oxygen per kilogram of body mass per minute).

**Supplementary Figure 1**.

**All PLCRC participants
(2013-Aug’19)**

PLCRC 2013-’16

PLCRC 2017-Aug’19

AU-ROC = 0.64

AU-ROC = 0.70

**AU-ROC = 0.65**

**Caption:** Area under the ROC curves (AU-ROC) as a predictive performance measure to evaluate the logistic regression models’ ability to discriminate between PLCRC participants and non-participants, based on age at diagnosis, sex, primary tumor location, and tumor stage.

**References**

1. Aaronson, N. K. *et al.* The European Organization for Research and Treatment of Cancer qlq-c30: A quality of life instrument for use in international clinical trials in oncology. *J. Natl. Cancer Inst.* **85**, 365–376 (1993).

2. Wendel-Vos, G. C., Schuit, A. J., Saris, W. H. & Kromhout, D. Reproducibility and relative validity of the short questionnaire to assess health-enhancing physical activity. *J. Clin. Epidemiol.* **56**, 1163–1169 (2003).

3. Smets, E. M., Garssen, B., Bonke, B. & De Haes, J. C. The Multidimensional Fatigue Inventory (MFI) psychometric qualities of an instrument to assess fatigue. *J. Psychosom. Res.* **39**, 315–325 (1995).

4. Sangha, O., Stucki, G., Liang, M. H., Fossel, A. H. & Katz, J. N. The Self-Administered Comorbidity Questionnaire: A new method to assess comorbidity for clinical and health services research. *Arthritis Rheum.* **49**, 156–163 (2003).

5. Purcell, A., Fleming, J., Bennett, S., Burmeister, B. & Haines, T. Determining the minimal clinically important difference criteria for the Multidimensional Fatigue Inventory in a radiotherapy population. *Support. Care Cancer* **18**, 307–315 (2010).

6. Osoba, D., Rodrigues, G., Myles, J., Zee, B. & Pater, J. Interpreting the significance of changes in health-related quality-of-life scores. *J. Clin. Oncol.* **16**, 139–144 (1998).

7. Giesinger, J. M. *et al.* Thresholds for clinical importance were established to improve interpretation of the EORTC QLQ-C30 in clinical practice and research. *J. Clin. Epidemiol.* **118**, 1–8 (2019).

8. Statistics Netherlands (Centraal Bureau voor de Statistiek - CBS). Gezondheidsenquete 2017 - GECON 2017. DANS. https ://doi.org/10.17026 /dans-xxd-j335 (2017).

9. Schwarz, R., Krauss, O. & Hinz, A. Fatigue in the general population. *Onkologie* **26**, 140–144 (2003).

10. van de Poll-Franse, L. V. *et al.* Normative data for the EORTC QLQ-C30 and EORTC-sexuality items in the general Dutch population. *Eur. J. Cancer* **47**, 667–675 (2011).

11. Weggemans, R. M. *et al.* The 2017 Dutch physical activity guidelines. *Int. J. Behav. Nutr. Phys. Act* **15**, 58 (2018).
